# The androgen receptor confers protection against diet-induced atherosclerosis, obesity, and dyslipidemia in female mice

**DOI:** 10.1096/fj.14-259234

**Published:** 2014-12-30

**Authors:** Johan B. Fagman, Anna S. Wilhelmson, Benedetta M. Motta, Carlo Pirazzi, Camilla Alexanderson, Karel De Gendt, Guido Verhoeven, Agneta Holmäng, Fredrik Anesten, John-Olov Jansson, Malin Levin, Jan Borén, Claes Ohlsson, Alexandra Krettek, Stefano Romeo, Åsa Tivesten

**Affiliations:** *Wallenberg Laboratory for Cardiovascular and Metabolic Research, Institute of Medicine, Sahlgrenska Academy, University of Gothenburg, Gothenburg, Sweden; ^†^Laboratory for Experimental Medicine and Endocrinology, Department of Experimental Medicine, Katholieke Universiteit Leuven, Leuven, Belgium; ^‡^Department of Physiology, Institute of Neuroscience and Physiology, and ^§^Centre for Bone and Arthritis Research, Institute of Medicine, Sahlgrenska Academy, University of Gothenburg, Gothenburg, Sweden; ^¶^Nordic School of Public Health, Gothenburg, Sweden; and ^‖^Department of Internal Medicine and Clinical Nutrition, Institute of Medicine, Sahlgrenska Academy, University of Gothenburg, Gothenburg, Sweden

**Keywords:** genetically altered mice, metabolism, sex hormones

## Abstract

Androgens have important cardiometabolic actions in males, but their metabolic role in females is unclear. To determine the physiologic androgen receptor (AR)–dependent actions of androgens on atherogenesis in female mice, we generated female AR-knockout (ARKO) mice on an atherosclerosis-prone apolipoprotein E (apoE)–deficient background. After 8 weeks on a high-fat diet, but not on a normal chow diet, atherosclerosis in aorta was increased in ARKO females (+59% *vs*. control apoE-deficient mice with intact AR gene). They also displayed increased body weight (+18%), body fat percentage (+62%), and hepatic triglyceride levels, reduced insulin sensitivity, and a marked atherogenic dyslipidemia (serum cholesterol, +52%). Differences in atherosclerosis, body weight, and lipid levels between ARKO and control mice were abolished in mice that were ovariectomized before puberty, consistent with a protective action of ovarian androgens mediated *via* the AR. Furthermore, the AR agonist dihydrotestosterone reduced atherosclerosis (−41%; thoracic aorta), subcutaneous fat mass (−44%), and cholesterol levels (−35%) in ovariectomized mice, reduced hepatocyte lipid accumulation in hepatoma cells *in vitro*, and regulated mRNA expression of hepatic genes pivotal for lipid homeostasis. In conclusion, we demonstrate that the AR protects against diet-induced atherosclerosis in female mice and propose that this is mediated by modulation of body composition and lipid metabolism.—Fagman, J. B., Wilhelmson, A. S., Motta, B. M., Pirazzi, C., Alexanderson, C., De Gendt, K., Verhoeven, G., Holmäng, A., Anesten, F., Jansson, J.-O., Levin, M., Borén, J., Ohlsson, C., Krettek, A., Romeo, S., Tivesten, A. The androgen receptor confers protection against diet-induced atherosclerosis, obesity, and dyslipidemia in female mice.

Circulating levels of testosterone, the main androgen, in females are approximately 1/10th of the levels in males. In women, androgens are secreted by both ovaries and adrenals, but in female mice, the ovaries are the dominant producers of androgens. In women, as in men, testosterone levels slowly decline with increasing age. Low androgen levels may also be caused, for example, by bilateral ovariectomy, hypopituitarism, and glucocorticosteroid use, whereas androgen levels are not significantly altered across menopause (1).

Substantial evidence from both human and rodent studies indicates that androgens exert important effects on atherosclerosis, body composition, and metabolism in males (2–5). By contrast, the physiologic metabolic actions of androgens in females are poorly understood (1), and the ongoing debate concerning the possible existence of an androgen deficiency syndrome in women has mainly focused on well-being and sexual function (6, 7). The coexistence of excess androgen production and a cluster of cardiovascular risk factors (*e.g.,* obesity, insulin resistance, and dyslipidemia), as well as increased atherosclerosis (8), in polycystic ovary syndrome has favored the idea that androgens exert adverse metabolic effects in women (9, 10).

Studies on the role of androgens in females are hampered by, for example, the concomitant loss of androgens and estrogens following ovariectomy and difficulties in assessing low serum levels of androgens in females, as well as determining appropriate doses for androgen replacement (1, 11). The physiologic effects of androgens are mostly mediated by activation of the ubiquitously expressed androgen receptor (AR) (12), and the development of female AR knockout (ARKO) mice has provided a valuable tool for studying the role of androgens in females (13).

To determine the physiologic AR-dependent actions of androgens on the development of atherosclerosis in females, we generated female ARKO and control mice on an atherosclerosis-prone apolipoprotein E (apoE)–deficient background.

## MATERIALS AND METHODS

### Animals

Because of the vital importance of the AR in male fertility, Cre/loxP technology (14) was used to obtain female ARKO and control mice on an atherosclerosis-prone apoE-deficient background (AR^−/−^apoE^−/−^ and AR^+/+^apoE^−/−^, respectively). Mice expressing Cre recombinase ubiquitously and from an early embryonic stage under the control of the phosphoglycerate kinase-1 promoter (PGK-Cre) (15) and mice with floxed AR exon 2 (floxAR) (14) were backcrossed to a C57BL/6 background (Charles River Laboratories) for ≥6 generations and then crossed with apoE^−/−^ (C57BL/6 background, model APOE-M; Taconic Europe A/S, Lille Skensved, Denmark) to obtain a homozygous apoE^−/−^ background. Because of the location of the AR on the X chromosome, the infertility of AR^−/Y^ males (14), and the fact that Cre activity starts in the diploid phase of oogenesis in PGK-Cre-expressing females and thereby causes complete recombination in Cre^-^ offspring (15), the breeding was performed as follows: AR^+/flox^apoE^−/−^ female mice and Cre^+/+^apoE^−/−^ male mice were crossed to obtain heterozygous AR^+/−^Cre^+/−^apoE^−/−^ female offspring. These AR^+/−^Cre^+/−^apoE^−/−^ females were mated with AR^flox/Y^apoE^−/−^ males to generate AR^−/−^apoE^−/−^ (ARKO) females. Female AR^+/+^apoE^−/−^ (control) mice were generated by mating AR^+/−^Cre^+/−^apoE^−/−^ females with AR^+/Y^apoE^−/−^ males. Because Cre expression itself did not affect the metabolic phenotype and there were similar effects on atherosclerosis by ARKO in Cre^+^ and Cre^−^ mice, Cre^+^ and Cre^−^ animals were pooled in both the ARKO and control groups. We assessed AR (14), Cre (14), and apoE (protocol from The Jackson Laboratory, Bar Harbor, ME, USA) genotypes using PCR amplification of genomic DNA from the tail.

### Study protocols

The mice were housed in a temperature- and humidity-controlled room with a 6:00 am to 6:00 pm light cycle and fed diet and tap water *ad libitum*. All procedures were approved by the Ethics Committee on Animal Care and Use in Gothenburg and conformed to the U.S. National Institutes of Health *Guide for the Care and Use of Laboratory Animals*.

#### Protocol 1: Gonadal-intact female ARKO and control mice

Female ARKO and control mice were fed a normal chow diet (NCD) after weaning and, in separate experiments, an NCD or a high-fat diet (HFD; #821424, 21% fat from lard, 0.15% cholesterol; Special Diets Services, Essex, United Kingdom) from 8 to 16 weeks of age. Some mice were kept on an NCD until 34 weeks of age.

#### Protocol 2: Ovariectomized female ARKO and control mice

Prepubertal female ARKO and control mice (23–25 days old) were anesthetized with isoflurane (Baxter Medical AB, Kista, Sweden) and bilaterally ovariectomized. From 8 to 16 weeks of age, the mice were fed an HFD as described in Protocol 1.

#### Protocol 3: Dihydrotestosterone treatment of ovariectomized female mice

Eight-week-old female apoE-deficient mice (Taconic Europe A/S) were bilaterally ovariectomized and implanted subcutaneously with a small slow-release pellet containing placebo or dihydrotestosterone (DHT) releasing 2.5 mg/90 days (Innovative Research of America, Sarasota, FL, USA). From 8 to 16 weeks of age, the mice were fed an HFD as described in Protocol 1.

#### Protocol 4: Orchiectomized male mice

Prepubertal male apoE-deficient mice (23–25 days old) were anesthetized with isoflurane and sham-operated or bilaterally orchiectomized. From 8 to 16 weeks of age, the mice were fed an HFD as described in Protocol 1. Total RNA was extracted from the liver as described below.

### *En face* analysis of the aorta

At the study end, the circulatory system was perfused with 0.9% saline (pH 7.4) under physiologic pressure. The entire aorta was dissected out from the heart to the iliac bifurcation and fixed in 4% paraformaldehyde. For *en face* analysis, the aortas were dissected free from connective and adipose tissue, cut open longitudinally, and pinned flat on silicone-coated dishes. The aortas were stained for lipids using Sudan IV, and images were captured. The outline of the aortic surface and lesions were defined manually by a blinded observer, and lesion areas were computed by an image analysis program (BioPix Software, Göteborg, Sweden). The extent of atherosclerosis was expressed as the percentage of the aortic surface covered by lesions. Results were calculated for the aortic arch (from the brachiocephalic trunk to the first intercostal arteries), the thoracic aorta (from the first to the last intercostal arteries), the abdominal aorta (from the last intercostal arteries to the aortic bifurcation), and the whole aorta, respectively.

### Lesion analyses in the aortic root

Serial 10 *μ*m cryosections were cut distally from the aortic root. Sections were stained (200, 400, and 600 *µ*m after the appearance of the aortic cusps) with Oil Red O (Sigma-Aldrich, St. Louis, MO, USA) and Masson´s trichrome (Accustain Trichrome Stains–Masson; from Sigma-Aldrich). For immunohistochemical staining of macrophages, aortic root cryosections (160 *μ*m from the aortic cusps) were incubated with rat anti-mouse Mac-2 antibody (1:1000; Cedarlane, Hornby, BC, Canada) or isotype control antibody (1:1000; Biolegend, San Diego, CA, USA), followed by horseradish peroxidase–conjugated goat anti-rat IgG secondary antibody (1:1000; GE Healthcare, London, United Kingdom) and visualized with the DAB substrate kit (Dako, Glostrup, Denmark). The areas of Oil red O staining, Mac-2 staining, and collagen staining (blue color in Masson’s trichrome) were determined using morphometric analysis (BioPix Software) and normalized to the size of the atherosclerotic lesion. Lesion complexity (presence/absence of necrotic core) was evaluated in sections stained with Masson´s trichrome (200 *μ*m from the aortic cusps).

### Dual energy X-ray absorptiometry

Body composition was assessed by dual energy X-ray absorptiometry (Lunar PIXImus 2 mouse densitometer; GE Lunar Corporation, Madison, WI, USA). The head region was excluded from analysis following the manufacturer’s recommendations. Lean, bone, and fat mass, as well as body fat percentage, were calculated by the Lunar PIXImus 2 2.10 software (GE Lunar Corporation).

### Serum analyses

All blood samples were collected from random-cycling mice after 3–4 hours of fasting. Blood was collected in serum gel tubes (Sarstedt, Nümbrecht, Germany), and serum was obtained and stored at −80°C. Serum levels of estradiol were analyzed using a radioimmunoassay (Siemens Healthcare Diagnostics, Tarrytown, NY, USA). Serum testosterone levels were measured by a radioimmunoassay after diethylether extraction (16). Levels of cholesterol, triglycerides, and serum glucose were analyzed by colorimetric assays using Infinity reagents (TR13421, TR22421, and TR15421; Thermo Fisher Scientific, Waltham, MA, USA). The distribution of lipids within the plasma lipoprotein fractions was assessed in pooled serum (6 mice per pool) by fast-performance liquid chromatography gel filtration using a Superose 6HR 10/30 column (Pharmacia, Uppsala, Sweden) (17). Insulin levels were measured using a mouse insulin ELISA (Ultra Sensitive Mouse Insulin ELISA; Crystal Chem, Downers Grove, IL, USA).

### Triglyceride content in liver

Liver samples were homogenized with isopropanol using stainless steel beads (5 mm; Qiagen, Hilden, Germany) in a TissueLyser II (Qiagen), kept for 1 hour at 4°C, and centrifuged for 5 minutes (2500 rpm, 4°C), and the supernatants were collected. The triglyceride levels in the supernatants were analyzed using an Infinity reagent (Triglycerides #TR22421; Thermo Fisher Scientific), and the amount of triglycerides was normalized to liver sample weights.

### Insulin and glucose tolerance tests

#### Insulin tolerance test

The mice were given an intraperitoneal injection (0.25 U/kg in mice fed a normal chow diet; 0.5 U/kg body weight in HFD-fed mice) of insulin (Actrapid; Novo Nordisk, Bagsværd, Denmark), and blood glucose was determined in serial blood samples from the tail vein using the HemoCue Glucose system (HemoCue, Ängelholm, Sweden). The mice were fasted prior to the insulin injection and during the measurements until the 120-minute time point.

#### Glucose tolerance test

The mice were given an intraperitoneal injection (2 mg/kg body weight in 0.9% saline) of d-glucose (Sigma-Aldrich), and blood glucose was determined in serial blood samples from the tail as described above. The mice were fasted prior to the injection of glucose and during the test.

### Blood pressure measurements

The mice were anesthetized with isoflurane (Baxter Medical AB), and a Samba transducer catheter (Samba Sensors, Gothenburg, Sweden) was placed into the left carotid artery for measurements of diastolic, systolic, and mean arterial pressure, as well as heart rate. We collected data using a PowerLab data acquisition unit together with LabChart software (ADInstruments, Sydney, Australia), averaged over a period of 2 minutes, following stabilization of the arterial pressure trace.

### Locomotor activity, food intake, body temperature, and indirect calorimetry

The spontaneous locomotor activity was measured for 4 hours using open-field 50 × 50 cm plexiglas cages fitted with infrared beams (Kungsbacka Mät och Reglerteknik AB, Fjärås, Sweden). Activity counts were recorded each time a mouse broke an infrared beam. Rectal body temperatures were recorded in anesthetized mice (isoflurane; Baxter Medical AB). Food intake was measured individually over 24 hours. Indirect calorimetry was analyzed at room temperature (21°C) using an indirect open circuit calorimeter (Somedic INCA; Somedic sales AB, Hörby, Sweden). Briefly, the mice were placed in the individual cages in the specifically designed calorimeter chambers with a 6:00 am to 6:00 pm light cycle and *ad libitum* access to food and water for 24 hours.

### Adipocyte size

Cryosections of mesenteric adipose tissue were stained with hematoxylin/eosin, and images were captured. The plasma membranes of individual adipocytes were defined manually by a blinded observer, and adipocyte surface areas were computed by an image analysis program (BioPix Software). An average of 300 adipocytes per mouse was analyzed.

### Cell culture and Oil red O staining

McArdle rat hepatoma cells (McA-RH 7777; LGC Standards AB, Borås, Sweden) were cultured in MEM (PAA Laboratories GmbH, Linz, Austria) containing 20% fetal bovine serum (HyClone Laboratories, South Logan, UT, USA). For assessment of intracellular lipids, the cells were plated in 24-well plates containing glass coverslips and grown in MEM with 2% fetal bovine serum. After 24 hours, the medium was changed to MEM without fetal bovine serum plus 0, 20, or 60 *µ*M oleic acid (Sigma-Aldrich), and after 48 hours, the cells were treated with 1 nM DHT (dissolved in ethanol; Sigma-Aldrich) or vehicle for 24 hours. In separate experiments, the medium was changed to MEM without fetal bovine serum plus 60 *µ*M oleic acid 24 hours after plating, and after 48 hours, the cells were treated with 1 nM DHT, 10 μM enzalutamide (Selleckchem, Houston, TX, USA), or vehicle for 24 hours. Cells were then fixed in 2% formaldehyde for 5 minutes, treated with 20% isopropanol for 0.5 minutes, and stained with Oil Red O for 20 minutes. Next, cells were treated with 20% isopropanol for 0.5 minutes, stained with hematoxylin for 2 minutes, and finally rinsed in tap and distilled water. Cells were photographed with a Zeiss microscope and Oil Red O–stained area per cell was quantified by BioPix iQ 2.1.8 software (BioPix Software).

### Triglyceride secretion analysis *in vitro*

Analysis of hepatic triglyceride secretion was performed as previously described (18). Briefly, McA-RH 7777 cells were grown in MEM with 2% fetal bovine serum. After 24 hours, the medium was changed to MEM without fetal bovine serum plus 20 *µ*M oleic acid, and after 48 h, DHT (10 nM) or vehicle was added for 24 hours. Cells were then incubated for 10 hours with [^3^H]glycerol (0.6 *μ*Ci/ml of culture medium; Perkin Elmer, Waltham, MA, USA) to trace triglycerides. Release of [^3^H]glycerol was chased in cold medium, and lipids were extracted in Folch solution (chloroform/methanol 2:1). Triglycerides were separated by thin layer chromatography on silica plates (Merck, Darmstadt, Germany) using a 2-phase system [first phase, chloroform:methanol:water (65:25:4 v/v); second phase, petroleumether:diethylether:acetic acid (80:20:1 v/v)], and [^3^H]glycerol was measured by scintillation counting (BeckMan Coulter, Fullerton, CA, USA).

### Apolipoprotein B secretion analysis *in vitro*

Apolipoprotein B secretion was analyzed as previously described (19). Briefly, McA-RH 7777 cells were incubated with MEM containing 2% fetal calf serum and 20 *μ*M oleic acid. After 24 hours, 10 nM DHT or vehicle was added for 24 hours. Next, cells were incubated for 2 h with methionine-free medium (Sigma-Aldrich), pulse-labeled for 20 min with [^35^S] methionine (Perkin Elmer), and chased with cold medium for 5, 15, 30, 60, and 120 minutes. Apolipoprotein B-100 and apolipoprotein B-48 were immunoprecipitated from the medium at each time point using a polyclonal anti-APOB apolipoprotein B antibody (DAKO, Glostrup, Denmark) and Pansorbin cells (Merck). SDS-PAGE was carried out overnight at +4°C, 24 mA, on a 5% polyacrylamide gel. The gel was exposed to a phosphor-screen, and bands were observed using Fuji FLA-3000 PhosphorImager and quantified using MultiGauge 2.0.

### mRNA expression in Liver

Total RNA was extracted from snap-frozen liver using RNeasy Mini Kit (Qiagen). cDNA was synthesized using the high capacity cDNA Reverse Transcription Kit (Applied Biosystems, Foster City, CA, USA) with random primers. mRNA expression of genes of interest was analyzed with TaqMan real-time PCR in an ABI Prism 7900 HT Detection System (Applied Biosystems). The following TaqMan Gene Expression assays were used: Ldlr mm00440169_m1; Vldlr mm00443281_m1; Pcsk9 mm00463738_m1; Scarb1 mm00450236_m1; Cpt1a mm00550438_m1; Mttp mm00435015_m1; Sort1 mm00490905_m1; ApoC3 mm00445670_m1; Nr1h3 (LXR*α*) mm00443451_m1; Srebf1 mm00550338_m1; and Srebf2 mm01306262_m1. 18S (Applied Biosystems) was used as an internal control.

### Statistical analyses

Statistical evaluations were performed with SPSS software (version 15.0; SPSS, Chicago, IL, USA). All variables were tested for normal distribution (Shapiro-Wilk normality test). For variables that were normally distributed with or without log transformation, 2 group comparisons were performed by Student’s *t* test and 4 group comparisons by 1-way ANOVA followed by Tukey’s *post hoc* test. Other (nonparametric) data were analyzed using a Mann-Whitney *U* test (2 groups). Frequencies were compared by χ^2^ test. *P* < 0.05 was considered statistically significant.

## RESULTS

### Increased atherosclerosis in female ARKO mice fed high-fat diet

**Figure 1.**
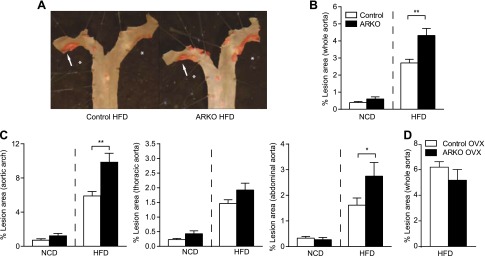
Increased atherosclerosis in female ARKO mice fed an HFD. Female control and ARKO mice on an apoE-deficient background were fed either an NCD or an HFD from 8 to 16 weeks of age (control NCD, *n* = 13; ARKO NCD, *n* = 11; control HFD, *n* = 21; ARKO HFD, *n* = 17). At the end of the study, atherosclerotic lesion area was assessed in aortas mounted *en face* and stained for lipids with Sudan IV. *A*) Representative photographs showing typically sized atherosclerotic lesions stained red (arrow) in the upper aorta of control and ARKO mice fed an HFD. *Inner curvature of the aorta. *B*, *C*) Atherosclerotic lesion area showing positive lipid staining with Sudan IV, expressed as the percentage of the total aortic surface area, in the whole aorta (*B*) and different segments of the aorta (*C*); *i.e.,* the aortic arch (from the brachiocephalic trunk to the first intercostal arteries), the thoracic aorta (from the first to the last intercostal arteries), and the abdominal aorta (from the last intercostal arteries to the aortic bifurcation). *D*) Atherosclerotic lesion area in female control (*n* = 9) and ARKO (*n* = 12) mice on an apoE-deficient background that were ovariectomized (OVX) before puberty and fed an HFD from 8 to 16 weeks of age. All values are provided as means ± sem. *P < 0.05 and **P < 0.01 *vs*. control HFD (*t* test on log-transformed values).

We evaluated atherosclerotic lesion formation in *en face*-mounted aortas of 16-week-old female control and ARKO mice on an apoE-deficient background fed either an NCD or an HFD from 8 weeks of age. Lesion area in the whole aorta, aortic arch, thoracic aorta, and abdominal aorta did not differ between ARKO and control females fed an NCD (**Fig. 1**). By contrast, after 8 weeks on HFD, the lesion area in the whole aorta was increased by 59% in ARKO compared with control mice (Fig. 1*A*, *B*). The lesion area was significantly increased in the aortic arch (+67%) and the abdominal aorta (+70%), but not in the thoracic aorta (Fig. 1*C*). There were no differences in plaque complexity (the presence/absence of a necrotic core) or composition (area of collagen and macrophage staining) in the aortic root between the genotypes in mice fed HFD (Supplemental Table S1).

Because endogenous androgens are derived solely from the ovaries in female mice (20), we postulated that the observed difference in atherosclerosis between ARKO and control mice fed an HFD would be abolished in ovariectomized mice. Indeed, we observed similar levels of atherosclerosis in HFD-fed ARKO and control mice that were ovariectomized before puberty (Fig. 1*D*).

We also evaluated endogenous hormone production in 16-week-old ovarian-intact mice fed an HFD. Serum estradiol levels were similar in ARKO and control mice, whereas serum testosterone levels were higher in female ARKO mice (Supplemental Table S2). The weights of uterus and ovaries did not differ between ARKO and controls (Supplemental Table S2).

### Diet-induced obesity in female ARKO mice

**Figure 2.**
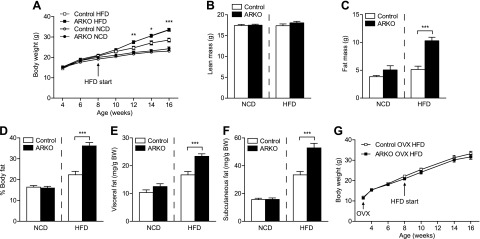
Diet-induced obesity in female ARKO mice. Female control and ARKO mice on apoE-deficient background were fed either an NCD or an HFD from 8 to 16 weeks of age. *A*) Body weights during the study. Lean mass (*B*), fat mass (*C*), and body fat percentage (*D*) analyzed by dual energy X-ray absorptiometry at 15 weeks of age (control NCD, *n* = 7; ARKO NCD, *n* = 7; control HFD, *n* = 12; ARKO HFD, *n* = 11). Weights of dissected visceral mesenteric (*E*) and subcutaneous inguinal fat depots (*F*) normalized to body weight (BW) at 16 weeks of age. (*G*) Body weight of female control (*n* = 9) and ARKO (*n* = 12) mice on an apoE-deficient background that were ovariectomized (OVX) before puberty and fed HFD from 8 to 16 weeks of age. Number of mice in each analysis (unless otherwise stated): control NCD, *n* = 13; ARKO NCD, *n* = 11; control HFD, *n* = 21; ARKO HFD, *n* = 17. All values are provided as means ± sem. *P < 0.05, **P < 0.01, and ***P < 0.001 *vs*. control HFD (Mann-Whitney test).

Between 4 and 16 weeks of age, body weight did not differ between ARKO and control females fed an NCD (**Fig. 2*A***). However, body weight was significantly higher in ARKO mice after 4 (+11%), 6, and 8 (+18%) weeks on an HFD (age 12, 14, and 16 weeks; Fig. 2*A*). Dual energy X-ray absorptiometry confirmed an increase in fat mass (+101%; Fig. 2*C*) and body fat percentage (+62%; Fig. 2*D*) without any changes in absolute bone mass (data not shown) or lean body mass (Fig. 2*B*) in HFD-fed ARKO mice. Further, the relative weights of dissected visceral and subcutaneous fat were increased in ARKO compared with control mice on an HFD (Fig. 2*E*, *F*). There were no differences in fat mass, body fat percentage, visceral fat, or subcutaneous fat between ARKO and control mice fed an NCD (Fig. 2*C–F*). Further, body weight (Fig. 2*G*) and visceral fat mass (25.1 ± 1.8 *vs*. 24.8 ± 0.7 mg/g body weight; *P* = not significant) were similar in HFD-fed ARKO and control mice that were ovariectomized before puberty. We also monitored body weight of ARKO and control females fed an NCD until 34 weeks of age and found no difference in body weight during this time (body weight at 34 weeks: ARKO 31.4 ± 1.6 *vs*. control 31.8 ± 2.1 g, *n* = 7/group; *P* = not significant). Despite the diet-induced obesity in female ARKO mice, we found no significant differences in locomotor activity, food intake, body temperature, oxygen consumption, or carbon dioxide production between ARKO and control females fed an HFD (Supplemental Fig. S1*A–E*).

### Diet-induced metabolic dysfunction including dyslipidemia, hepatic steatosis, and insulin resistance in female ARKO mice

**Figure 3.**
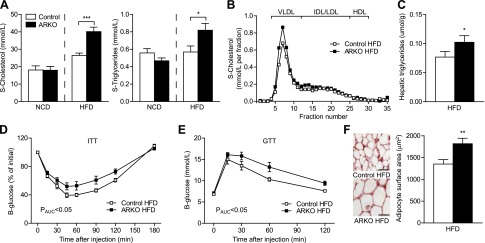
Diet-induced metabolic dysfunction including dyslipidemia, hepatic steatosis, and insulin resistance in female ARKO mice. Female control and ARKO mice on apoE-deficient background were fed either an NCD or an HFD from 8 to 16 weeks of age. *A*) Serum levels of total cholesterol and triglycerides at 16 weeks (control NCD, *n* = 7; ARKO NCD, *n* = 6; control HFD, *n* = 10; ARKO HFD, *n* = 11). *P < 0.05 and ***P < 0.001 *vs*. control HFD (*t* test). *B*) Cholesterol distribution in lipoprotein fractions at 16 weeks (control HFD and ARKO HFD; *n* = 6 mice per pool). *C*) Triglyceride content in the liver at 16 weeks (control HFD, *n* = 21; ARKO HFD, *n* = 17). *P < 0.05 *vs*. control HFD (*t* test on log-transformed values). *D*) Intraperitoneal insulin tolerance test at 14 weeks of age; blood glucose levels are expressed as percentage of initial values (control HFD, *n* = 12; ARKO HFD, *n* = 11). *P* value by Mann-Whitney test. *E*) Intraperitoneal glucose tolerance test at 14 weeks of age (control HFD and ARKO HFD; *n* = 6/group). *P* value by Mann-Whitney test. *F*) Mean adipocyte size in mesenteric adipose tissue at 16 weeks (control HFD, *n* = 21; ARKO HFD, *n* = 17). Scale bars = 50 *μ*m. **P < 0.01 *vs*. control HFD (*t* test on log-transformed values). All values are provided as means ± sem.

We next investigated whether there were differences in serum lipid levels between female ARKO and control mice fed an HFD. Indeed, serum total cholesterol levels were elevated by 52% and triglyceride levels by 44% in ARKO *versus* control mice after 8 weeks on an HFD (**Fig. 3*A***), with a similar lipid distribution in lipoprotein fractions in the groups (Fig. 3*B*). Further, hepatic triglyceride levels were increased in ARKO mice (Fig. 3*C*). Both insulin and glucose tolerance were significantly impaired in ARKO compared with control females fed an HFD (Fig. 3*D*, *E*). In line with an insulin-resistant phenotype, ARKO mice displayed enlarged mesenteric adipocytes after 8 weeks of an HFD (Fig. 3*F*). In mice fed an NCD, there were no differences in either serum cholesterol and triglycerides (Fig. 3*A*) or insulin and glucose tolerance (Supplemental Fig. S2*A*, *B*). Further, HFD-fed ARKO and control mice that were ovariectomized before puberty displayed no differences in serum cholesterol or triglycerides (Supplemental Fig. S2*C*). Blood pressure did not differ between ARKO and control mice fed HFD (Supplemental Table S3).

### Treatment with an AR agonist reduces atherosclerosis, subcutaneous fat mass, and cholesterol levels in ovariectomized female mice

**Figure 4.**
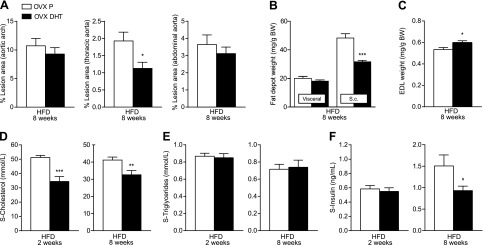
The AR agonist DHT reduces atherosclerosis, subcutaneous fat mass, and cholesterol levels in ovariectomized female mice. Female apoE-deficient mice were ovariectomized (OVX) and treated with the AR agonist DHT or placebo (P) from 8 weeks of age and fed an HFD between 8 and 16 weeks of age. *A*) Atherosclerotic lesion area in the aortic arch, thoracic, and abdominal aorta after lipid staining with Sudan IV, expressed as percentage of the total area (*n* = 15/group) at 16 weeks. *P < 0.05 *vs*. OVX P (*t* test on log-transformed values). *B*) Weights of dissected visceral and subcutaneous (s.c.) fat depots normalized to body weight (BW) (OVX P, *n* = 14; OVX DHT, *n* = 16). ***P < 0.001 *vs*. OVX P (Mann-Whitney test). *C*) Weight of dissected skeletal muscle [extensor digitorum longus (EDL)] normalized to BW (OVX P, *n* = 14; OVX DHT, *n* = 16). *P < 0.05 *vs*. OVX P (*t* test). *D*) Serum levels of total cholesterol after 2 and 8 weeks of HFD and DHT treatment (*n* = 15/group). **P < 0.01 and ***P < 0.001 *vs*. OVX P (Mann-Whitney test). *E*) Serum levels of triglycerides after 2 and 8 weeks of HFD and DHT treatment (*n* = 15/group). *F*) Serum insulin after 2 and 8 weeks (*n* = 14/group). *P < 0.05 *vs*. OVX P (*t* test on log-transformed values). All values are provided as means ± sem.

To confirm an atheroprotective action that is mediated *via* the AR in females, ovariectomized apoE-deficient mice were treated with the nonaromatizable AR agonist DHT or placebo for 8 weeks while simultaneously receiving an HFD. DHT treatment reduced *en face* atherosclerotic lesion area in the thoracic aorta by 41% compared with placebo, whereas there was no significant effect in the aortic arch or the abdominal aorta (**Fig. 4*A***).

DHT also reduced the relative weight of subcutaneous (−44%) but not visceral fat (Fig. 4*B*). Further, DHT increased skeletal muscle mass (Fig. 4*C*), and final body weight was not significantly different in DHT- *versus* placebo-treated mice (31.8 ± 0.9 *vs*. 29.7 ± 0.8 g, *P* = not significant).

After both 2 and 8 weeks of an HFD, DHT decreased serum total cholesterol levels (by 35% and 26%, respectively; Fig. 4*D*). By contrast, DHT did not affect serum triglyceride levels during the study (Fig. 4*E*). Moreover, DHT treatment reduced fasting serum insulin levels after 8 weeks but not 2 weeks (Fig. 4*F*). Blood glucose levels were unchanged at both time points (data not shown).

### AR agonist regulation of hepatic lipid metabolism in female mice

**Figure 5.**
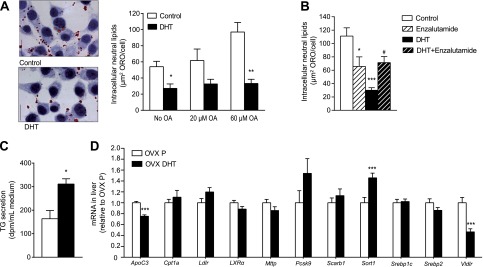
The AR agonist DHT regulates hepatic lipid metabolism in hepatoma cells *in vitro* and in female mice *in vivo*. *A*) McA RH-7777 cells were exposed to various concentrations of oleic acid (OA) followed by DHT (1 nM) or vehicle for 24 hours, and intracellular lipids were stained by Oil Red O (ORO; red). Scale bars = 10 *μ*m. Data are from 2 experiments. *P < 0.05 and **P < 0.01 *vs*. control (*t* test). *B*) McA RH-7777 cells were exposed to 60 *μ*M OA followed by DHT, enzalutamide (10 *μ*M), or vehicle for 24 hours, and intracellular lipids were stained by ORO. Data are from 4 experiments. *P < 0.05 and ***P < 0.001 *vs*. control; ^#^*P* < 0.05 *vs*. DHT (ANOVA followed by Tukeys *post hoc* test). *C*) For analysis of triglyceride secretion, McA RH-7777 cells were exposed to 20 *μ*M OA and DHT or vehicle and incubated with [^3^H]glycerol to trace triglycerides. Release of [^3^H]glycerol was chased in cold medium, lipids were extracted, and triglycerides were separated by thin layer chromatography. Data are from 1 experiment and expressed as disintegrations per minute per milliliter medium. *P < 0.05 *vs*. control (*t* test). *D*) mRNA expression of genes involved in the regulation of lipid metabolism was analyzed in livers of OVX female mice fed an HFD and treated with DHT or P for 8 weeks (*n* = 15–16/group). ***P < 0.001 *vs*. OVX P (*t* test; sort1 log-transformed). *ApoC3*, apolipoprotein CIII; *Cpt1a*, carnitine palmitoyltransferase Ia; *Ldlr*, low-density lipoprotein receptor; *LXRα*, liver X receptor *α*; *Mttp*, microsomal triglyceride transfer protein; *Pcsk9*, proprotein convertase subtilisin/kexin type 9; *Scarb1*, scavenger receptor class B, member 1; *Sort1*, sortilin 1; *Srebp1c*, sterol regulatory element-binding protein 1c; *Srebp2*, sterol regulatory element-binding protein 2; *Vldlr*, very low-density lipoprotein receptor. All values are provided as means ± sem.

Dyslipidemia is the most important risk factor leading to the development of atherosclerosis (21, 22) and is tightly coupled to alterations in hepatic lipid metabolism. To study potential direct effects of androgens on hepatic lipid accumulation, we incubated McA RH-7777 hepatoma cells with DHT. We found that DHT significantly reduced neutral lipid storage in hepatocytes (**Fig. 5*A***). This effect was blocked by concomitant treatment with the AR blocker enzalutamide (Fig 5*B*).

As a major pathway for removal of hepatic lipids is the incorporation of triglycerides into very low-density lipoproteins (VLDLs) and subsequent secretion, we hypothesized that DHT may affect this pathway. We found an increase in the amount of secreted triglycerides (Fig. 5*C*) with no difference in the amount of apoB secreted from the cells (Supplemental Fig. S3).

We also measured levels of selected mRNAs that encode proteins involved in the regulation of lipid metabolism in the livers of ovariectomized apoE-deficient female mice fed an HFD and treated with DHT or placebo. Of 11 studied mRNAs, 3 were regulated by DHT: mRNA levels of *ApoC3* (−25%) and *Vldlr* (−53%) were decreased and mRNA levels of *Sort1* were increased (46%; Fig. 5*D*). To study whether these genes are androgen regulated in males as in females, we analyzed mRNAs in the livers of intact and orchiectomized apoE-deficient male mice fed an HFD. Although *ApoC3* mRNA (+11%, *P* = not significant) was not regulated by castration in males, the castrated males showed a decrease in *Sort1* mRNA (−26%, *P* < 0.001 by *t* test) and a strong increase in *Vldlr* mRNA (+784%, *P* < 0.001 by *t* test), in accordance with the results from DHT treatment in females.

## DISCUSSION

The physiologic metabolic actions of androgens in females are poorly understood. The current study provides clear evidence that deletion of the AR in atherosclerosis-prone apoE-deficient female mice promotes the development of atherosclerosis, as well as obesity and dyslipidemia.

This is the first study to investigate atherogenesis in an animal model of female AR deficiency. Earlier studies to elucidate the effect of exogenous androgens on atherosclerosis in females report conflicting results (23–28). An often-cited study reported that testosterone treatment of ovarian-intact female monkeys caused increased atherosclerosis (25); however, the treatment completely suppressed ovarian cyclicity, suggesting that a disturbed estrogen production may contribute to these results. By contrast, a number of studies in ovariectomized female mice showed atheroprotection by testosterone (27, 28), and similar to our findings, 1 study showed that the nonaromatizable AR agonist DHT protected against atherosclerosis in female apoE-deficient mice (26), supporting the importance of the AR. However, in all these earlier studies, pharmacologic doses of androgens were administered exogenously, and thus these studies do not provide information on the physiologic effects of endogenous androgens.

In the present study, female AR deficiency resulted in increased atherosclerosis in the aortic arch and abdominal aorta with no significant difference in the thoracic aorta, whereas DHT treatment reduced atherosclerosis in the thoracic aorta only. We speculate that this may be explained by regional differences in the kinetics of lesion development. It is well known that lesions appear first in the aortic arch and abdominal aorta in the apoE-deficient mouse model (29). Therefore, these areas may be more susceptible to factors operating early during atherogenesis (*e.g.,* AR deficiency from embryonic stage) but less susceptible to atheroprotective factors operating in adult age (such as DHT treatment in this study) when complex atherosclerotic lesions have already developed in the aortic arch and abdominal aorta.

Hypercholesterolemia is the most important risk factor for atherosclerosis and associated complications (21, 22). In the present study, we found that AR deficiency increased serum lipid levels and atherosclerosis in female mice, whereas AR stimulation exerted the opposite actions. Dyslipidemia in female mice with general AR deficiency has not been addressed previously. In accordance with our data, previous studies report a reduction of serum lipid levels by exogenous androgens to both ovariectomized female mice (28, 30) and women (31). Limited available data do not support beneficial effects of testosterone treatment on lipid profiles in postmenopausal women (32); however, dose and mode of administration, as well as background endogenous hormone production, may determine treatment effects (1, 32).

It seems likely that the diet-induced obesity and associated metabolic complications, such as hepatic fat accumulation, contribute to an atherogenic dyslipidemia in female ARKO mice (22). Using an *in vitro* model, we also show direct hepatic actions of a nonaromatizable AR agonist protecting hepatoma cells from lipid accumulation, in line with previous reports (33). Interestingly, serum cholesterol levels were reduced shortly after the initiation of AR agonist treatment to female mice *in vivo*, and we observed hepatic regulation of genes pivotal for lipid homeostasis, encoding sortilin, VLDL receptor (VLDLr), and apolipoprotein C3 (apoC3), in these mice. In accordance, we found that *Sort1* and *Vldlr*, but not *ApoC3* mRNAs were regulated by endogenous androgens (castration) in male mice. Liver-specific sortilin overexpression may dramatically reduce cholesterol levels in mice, but basic studies report conflicting results on sortilin action (34). The VLDLr mediates reuptake of VLDL into the liver, and downregulation of *Vldlr* may therefore contribute to protection from hepatic steatosis by androgens *in vivo* (35), although this mechanism may be less relevant in our apoE-deficient model. Notably, DHT treatment to females *in vivo* downregulated *ApoC3*, a major component of triglyceride-rich lipoproteins that reduces clearance of triglyceride-rich lipoproteins and is causally associated with atherogenesis (36, 37). Although the regulation of *ApoC3* mRNA was relatively modest (−25%) in our study, reduction of *ApoC3* mRNA in this range was recently shown to effectively reduce lipid levels in mice (37). Taken together, we propose that androgens reduce hepatic lipid accumulation by favoring a higher lipidation of VLDL with no changes in the total amount of secreted VLDL particles, as shown here *in vitro.* These particles are in turn more effectively cleared from the circulation due to a relative decrease in apoC3. In accordance with this notion, testosterone supplementation increased VLDL clearance in female apoE3-Leiden mice (30). The clinical relevance of these data is supported by a study suggesting that testosterone supplementation may improve lipid profiles in ovariectomized women by decreasing the apoC3 concentration in lipoproteins (31).

Our present results demonstrate that ARKO female mice display obesity when fed an HFD, but not an NCD. This finding is in line with earlier studies that reported late-onset obesity and metabolic dysfunction in male (38, 39) but not female (38, 40–42) ARKO mice fed an NCD. It is therefore clear that an HFD is required for the obese and metabolic phenotype in female ARKO mice, and possibly the apoE-deficient background of our mice and/or the lard content of the HFD further contributes to unmasking of the phenotype. In comparison, we previously found increased atherosclerosis but less pronounced alterations in serum lipid levels and lower body weight in male apoE-deficient ARKO mice fed an HFD (5); these discrepancies may be partly explained by developmental defects in male ARKO mice (43).

It remains a topic for future studies to determine the target organs for the metabolic effects of androgens in females. Supporting the notion of the liver as a major target organ, male mice with liver-specific inactivation of the AR [hepatic androgen receptor knockout (H-ARKO)] develop diet-induced obesity, insulin resistance, hepatic steatosis, and dyslipidemia (44). Although the latter study reported no similar phenotype in female H-ARKO, it is conceivable that adjustment of experimental conditions (diet, mouse background) may reveal metabolic disturbances also in female H-ARKO. Androgens may also exert direct AR-dependent effects on adipocyte differentiation and function (45), and hyperinsulinemia and visceral obesity are reported in male mice lacking the AR in adipose tissue (46), although others found no similar phenotypes (47). In the present study, the weights of both visceral and subcutaneous fat depots were increased in ARKO females, whereas the AR agonist reduced the weight of subcutaneous, but not visceral, fat. Androgens may affect adipocyte biology in a depot-specific manner (45), and several factors that differ between the two models (endogenous AR deficiency from embryonic stage *vs*. exogenous AR stimulation in adulthood, respectively) could potentially explain fat depot specificity. Irrespective of mechanism, this discrepancy may indirectly support a role of AR actions at the adipose tissue level.

In agreement with previous results (13), we found unchanged serum estradiol levels in female ARKO mice. Further, uterus weight was unchanged, indicating that peripheral estrogen action is not disturbed in ARKO females. Our finding of increased testosterone levels may suggest involvement of the AR in the feedback regulation of androgen production in females, as shown previously in males (48). The increased testosterone levels are unlikely to contribute to the increased atherosclerosis in the ARKO females; testosterone should have mainly estrogenic effects in the absence of the AR (12), and exogenous testosterone reduces atherosclerosis in apoE-deficient females (28). We also found that the observed difference in atherosclerosis between ARKO and control mice was abolished in ovariectomized mice. This result is consistent with a protective action of endogenous androgens that is mediated *via* the AR, given that ovariectomy results in complete loss of endogenous androgens in female mice and that estrogen levels were unchanged in ARKO females.

The main evidence in support of androgens exerting adverse metabolic effects in women is the fact that women with polycystic ovary syndrome (affecting 6–10% of all women) display both hyperandrogenism and an adverse metabolic risk profile with insulin resistance, obesity, dyslipidemia, and increased atherosclerosis (8–10). However, most evidence suggests that insulin resistance is central to the pathogenesis of polycystic ovary syndrome and that the androgen excess is secondary to compensatory hyperinsulinemia (49). In line with our present results, several studies also report an association between low androgen levels and atherosclerosis in women (50–53). Further, recent clinical studies have reported a U-shaped (54, 55) or an inverse (56, 57) association between androgen levels and cardiometabolic risk, consistent with a protective action by endogenous androgens in women. In comparison, low androgen levels have been associated with both increased atherosclerosis and cardiometabolic risk in men (3, 4).

To date, a definition of androgen deficiency in women is lacking (1, 6). Consequently, the symptoms, signs, and clinical outcomes associated with androgen deficiency are not well characterized (1, 6), and the clinical recommendation regarding testosterone supplementation to women is an area of disagreement (7). The results of the present study highlight the need for further studies on the cardiometabolic consequences of androgen deficiency and supplementation in women.

In conclusion, we demonstrate that the AR confers protection against diet-induced atherosclerosis in female mice and propose that this is mediated by modulation of body composition and lipid metabolism. Our results provide evidence that physiologic AR-dependent actions of androgens play a central metabolic role in females.

## Supplementary Material

Supplemental Data

## Abbreviations

apoC3: apolipoprotein C3
apoE: apolipoprotein E
AR: androgen receptor
ARKO: androgen receptor knockout
DHT: dihydrotestosterone
H-ARKO: hepatic androgen receptor knockout
HFD: high-fat diet
NCD: normal chow diet
VLDL: very low-density lipoprotein
VLDLr: VLDL receptor
