# Supplementary material for: The androgen receptor confers protection against diet-induced atherosclerosis, obesity, and dyslipidemia in female mice
Source: FASEB J. 2014 Dec 30;29(4):1540–50. doi: 10.1096/fj.14-259234 (PMC4470404; doi:10.1096/fj.14-259234)
Supplement: Supplemental Data [file supp_29_4_1540__index.html]

The androgen receptor confers protection against diet-induced atherosclerosis, obesity, and dyslipidemia in female mice — The androgen receptor confers protection against diet-induced atherosclerosis, obesity, and dyslipidemia in female mice — Supplemental Data 

# The androgen receptor confers protection against diet-induced atherosclerosis, obesity, and dyslipidemia in female mice

## Supplemental Data

**Files in this Data Supplement:**

- Supplemental Data
- Supplemental Data
