# Supplementary material for: The androgen receptor confers protection against diet-induced atherosclerosis, obesity, and dyslipidemia in female mice
Source: FASEB J. 2014 Dec 30;29(4):1540–50. doi: 10.1096/fj.14-259234 (PMC4470404; doi:10.1096/fj.14-259234)
Supplement: Supplemental Data [file supp_fj.14-259234_Supplemental_Data.pdf]

SUPPLEMENTAL TABLES

Table S1. Composition of aortic root atherosclerotic lesions in control and ARKO female mice

	Control HFD	ARKO HFD
Presence of a necrotic core (*n*; % of mice)	10/21; 48	7/16; 44
Collagen, stained area (% of plaque area)	31 ± 2	27 ± 3
Macrophages, stained area (% of plaque area)	20 ± 1	22 ± 2
Neutral lipids, stained area (% of plaque area)	29 ± 1	31 ± 2

Female control (*n* = 21) and ARKO (*n* = 16) mice on an apoE-deficient background were fed a high-fat diet (HFD) from 8 weeks of age until the study end (16 weeks of age), when cryosections from the aortic root were analyzed. Presence of a necrotic core was evaluated in sections stained with Masson´s trichrome. Collagen content was determined after staining with Masson´s trichrome, macrophage content after immunostaining for Mac-2 and neutral lipid after Oil Red O staining. Values are provided as means ± s.e.m, unless otherwise stated.

Table S2. Hormone levels and weights of reproductive organs in control and ARKO female mice

	Control HFD	ARKO HFD
Serum testosterone (ng/dL)	19.1 ± 1.2 (*n* = 9)	30.4 ± 2.3 (*n* = 10)*
Serum estradiol (pg/mL)	5.8 ± 1.4 (*n* = 7)	7.8 ± 2.0 (*n* = 7)
Uterus (mg)	99.8 ± 6.2 (*n* = 21)	99.7 ± 5.7 (*n* = 17)
Ovaries (mg)	7.6 ± 0.4 (*n* = 21)	7.4 ± 0.3 (*n* = 17)

Hormone levels and organ weights of 16-week-old random-cycling female control and ARKO mice on an apoE-deficient background after 8 weeks on a high-fat diet (HFD). All values are provided as means ± s.e.m. *P<0.001 (*t* test)

Table S3. Blood pressure and heart rate in control and ARKO female mice

	Control HFD	ARKO HFD
Systolic blood pressure (mmHg)	123 ± 2	125 ± 5
Diastolic blood pressure (mmHg)	88 ± 1	89 ± 3
Mean arterial blood pressure (mmHg)	100 ± 1	101 ± 4
Heart rate (beats/min)	462 ± 8	452 ± 8

Blood pressure and heart rate were measured invasively in 16-week-old female control (n=20) and ARKO mice (n=16) on an apoE-deficient background after 8 weeks on a high-fat diet (HFD). All values are provided as means ± s.e.m.

Supplemental Fig. 1

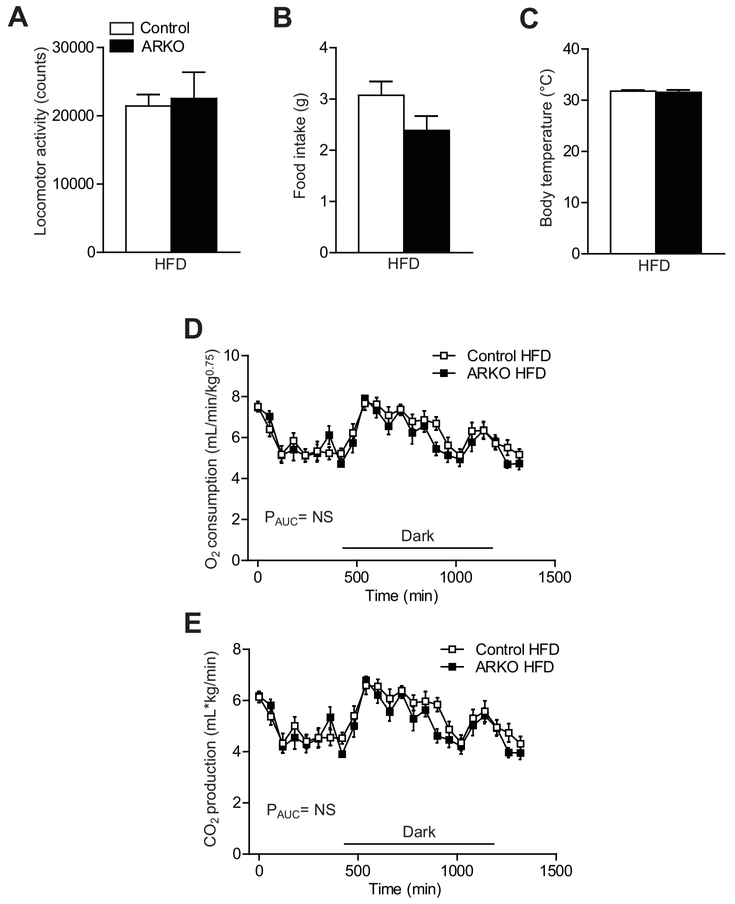

Fig. S1. Female control and ARKO mice on apoE-deficient background were fed a high-fat diet (HFD) from 8 to 16 weeks of age. (**A**) Spontaneous locomotor activity measured during 4 hours at 11 weeks of age (n=6/group). (**B**) Individual food intake during 24 hours at 14 weeks of age (n = 6/group). (**C**) Rectal body temperature at 15 weeks of age (n = 13-14/group). (**D**) Oxygen consumption and (**E**) Carbon dioxide production at 10 weeks of age (n = 8/group). All values are provided as means ± s.e.m.
