# Supplementary material for: The androgen receptor confers protection against diet-induced atherosclerosis, obesity, and dyslipidemia in female mice
Source: FASEB J. 2014 Dec 30;29(4):1540–50. doi: 10.1096/fj.14-259234 (PMC4470404; doi:10.1096/fj.14-259234)
Supplement: Supplemental Data [file supp_fj.14-259234_Supplemental_Figures.pdf]

Supplemental Fig. 2

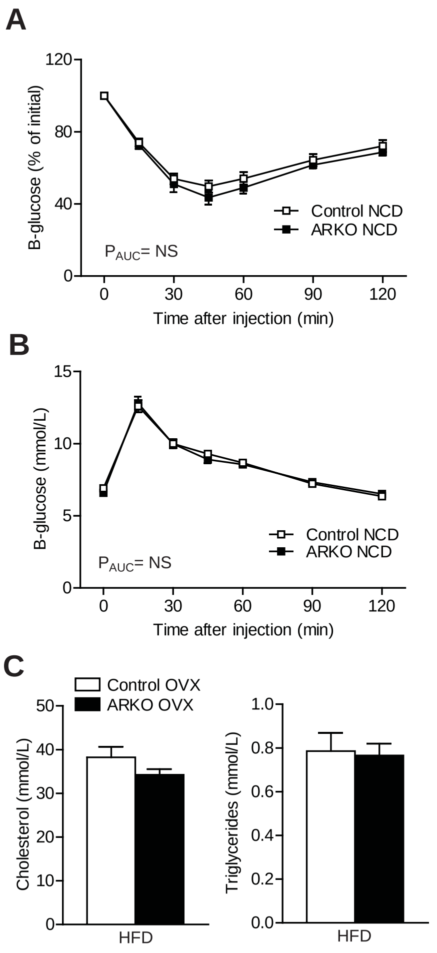

Fig. S2. (**A-B**) Female control and ARKO mice on apoE-deficient background fed normal chow diet (NCD). (**A**) Intraperitoneal insulin tolerance test (ITT) at 10 weeks of age; blood glucose levels are expressed as percentage of initial values (n = 13/group). (**B**). Intraperitoneal glucose tolerance test (GTT) at 12 weeks of age (n = 10-11/group). (**C**) Serum levels of total cholesterol and triglycerides in 16-week-old female control and ARKO mice on apoE-deficient background that were ovariectomized (OVX) before puberty and fed HFD from 8 to 16 weeks of age. (Control OVX HFD, n = 9; ARKO OVX HFD, n = 12). All values are provided as means ± s.e.m.

Supplemental Fig. 3

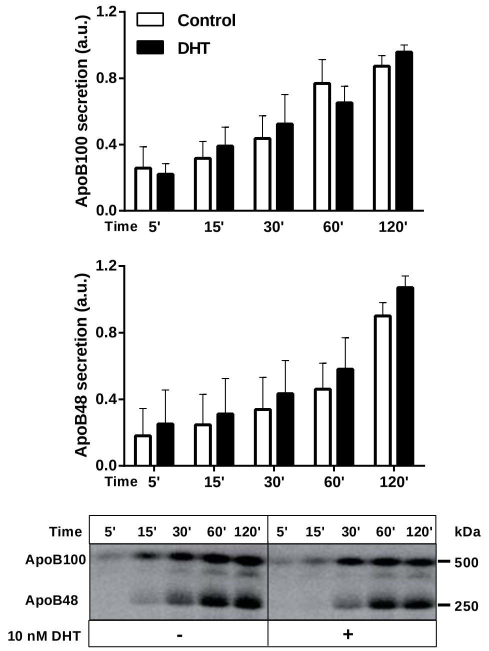

Fig. S3. DHT treatment does not affect apoB100 or apoB48 secretion in McA-RH 7777 cells. McA-RH 7777 cells were treated with 10 nM DHT and pulse-chased with [^{35}S]-methionine. ApoB100 and apoB48 were immunoprecipitated from media and visualized by Phosphorimager after separation on a SDS–PAGE. Bands were quantified by Multi Gauge Software. Each bar represents mean and SEM of 4 independent experiments. Values are expressed as a fraction of the most intense band from each experiment, which was arbitrarily set to 1. Abbreviations: DHT, Dihydrotestosterone; a.u., arbitrary units; kDa, kilo-Dalton.
